# Alzheimer's and Parkinson's disease therapies in the clinic

**DOI:** 10.1002/btm2.10367

**Published:** 2022-08-03

**Authors:** Puja Chopade, Neha Chopade, Zongmin Zhao, Samir Mitragotri, Rick Liao, Vineeth Chandran Suja

**Affiliations:** ^1^ Bob Jones High School Madison Alabama USA; ^2^ Department of Pharmaceutical Sciences, College of Pharmacy University of Illinois at Chicago Chicago Illinois USA; ^3^ School of Engineering and Applied Sciences Harvard University Cambridge Massachusetts USA; ^4^ Wyss Institute for Biologically Inspired Engineering Cambridge Massachusetts USA

**Keywords:** Alzheimer's Disease, clinical trials, delivery vehicles, drug administration routes, outcome measures, Parkinson's Disease, therapy mechanisms

## Abstract

Alzheimer's disease (AD) and Parkinson's disease (PD) are the most prevalent neurodegenerative diseases, affecting millions and costing billions each year in the United States alone. Despite tremendous progress in developing therapeutics that manage the symptoms of these two diseases, the scientific community has yet to develop a treatment that effectively slows down, inhibits, or cures neurodegeneration. To gain a better understanding of the current therapeutic frontier for the treatment of AD and PD, we provide a review on past and present therapeutic strategies for these two major neurodegenerative disorders in the clinical trial process. We briefly recap currently US Food and Drug Administration‐approved therapies, and then explore trends in clinical trials across the variables of therapy mechanism of disease intervention, administration route, use of delivery vehicle, and outcome measures, across the clinical phases over time for “Drug” and “Biologic” therapeutics. We then present the success rate of past clinical trials and analyze the intersections in therapeutic approaches for AD and PD, revealing the shift in clinical trials away from therapies targeting neurotransmitter systems that provide symptomatic relief, and towards anti‐aggregation, anti‐inflammatory, anti‐oxidant, and regeneration strategies that aim to inhibit the root causes of disease progression. We also highlight the evolving distribution of the types of “Biologic” therapies investigated, and the slowly increasing yet still severe under‐utilization of delivery vehicles for AD and PD therapeutics. We then briefly discuss novel preclinical strategies for treating AD and PD. Overall, this review aims to provide a succinct overview of the clinical landscape of AD and PD therapies to better understand the field's therapeutic strategy in the past and the field's evolution in approach to the present, to better inform how to effectively treat AD and PD in the future.

## 1 INTRODUCTION

Alzheimer's disease (AD) and Parkinson's disease (PD) are the two most common neurodegenerative disorders. AD and PD afflict around 6 million and 1 million patients, respectively, in the United States alone,^1^, ^2^ placing heavy financial and emotional burdens on patients as well as their caregivers. Between direct medical costs and indirect costs such as caregiver burden and disability income, AD and PD cost around $305 billion and $51.9 billion, respectively, in the United States annually.^2^, ^3^ Furthermore, as the population grow and lifespans increase, neurodegenerative diseases will become more widespread. Globally, AD and PD currently afflict around 50 million and 10 million people, respectively, and are projected to increase to 150 million and 12 million people, respectively, by the year 2050.^1^, ^4^, ^5^ Despite decades of research and investment, there are no clinically approved therapies that slow down or prevent the progression of these diseases. Instead, available treatments only mitigate symptoms. However, many experimental therapies have succeeded in preclinical animal models and are currently under investigation in clinical trials.

The US Food and Drug Administration (FDA) made the clinicaltrials.gov database publicly available in 2000 to document and display information about currently ongoing and completed clinical trials.^6^ This database is aimed towards helping patients, health care professionals, researchers, and the public easily access clinical studies for any disease. Patients are able to explore and register for clinical trials that involve therapies targeting the health conditions they are facing. The clinicaltrials.gov database is the largest registry of its kind and contains a comprehensive list of details for each clinical trial including a brief summary of the study as well as a description of the study design, aims and interventions, key outcome measures, and eligibility criteria for the trial. Its scope was expanded greatly in 2007 when all non‐phase 1 interventional clinical trials were required to report results and adverse effects to the database.^6^ Despite its value to patients seeking entry into specific trials investigating experimental therapies, the clinicaltrials.gov database lacks a concise cumulative display of information for researchers to glean understanding of the current state of the clinical frontier for a given disease or disorder.

To address this limitation, here we report the clinical trial frontier for the two major neurological disorders: Alzheimer's disease and Parkinson's disease. We begin by first summarizing current clinically approved therapies. To gain a better understanding of the clinical landscape of potential upcoming AD and PD treatments, we have sorted through AD and PD clinical trials to succinctly summarize the therapeutic intervention mechanism, administration route, delivery vehicle, and outcome measures of these investigations across clinical phases and therapy types for past and active registered interventional trials. We provide analyses that present the success rate of past clinical trials and highlight the directions and trends in the clinical landscape across past and active clinical trials. We finally close with a critical analysis of clinical intersections in therapeutic intervention strategies and a brief discussion of novel preclinical strategies for these two most prevalent neurodegenerative diseases. This analysis also emphasizes how the field can benefit from more widely adopting advanced drug delivery strategies utilizing delivery vehicles such as nanoparticle or cellular carriers to enhance therapeutic delivery and hence efficacy. The rest of the article is organized as follows. Section 2 details the approved therapies, Section 3 details the methodology followed for selecting the clinical trial data, Section 4 details the clinical landscape of AD therapies, Section 5 details the clinical landscape of PD therapies, Section 6 details the clinical intersections between AD and PD therapies, and Section 7 discusses novel preclinical strategies. Finally, we conclude the article in Section 8 detailing the future prospects in the field.

## 2 CURRENTLY APPROVED THERAPIES

### 2.1 Alzheimer's disease

AD is the most common neurodegenerative disorder that affects cognitive function, leading to dementia, confusion, and general mental decline. Classic AD symptoms occur due to brain atrophy and disrupted neuronal signaling in areas essential for cognition and memory, such as the hippocampus and cerebral cortex.^7^ AD is characterized at the cellular level by the accumulation of two types of insoluble protein aggregates throughout the brain: extracellular amyloid‐beta (Aβ) plaques and intraneuronal and extracellular tau neurofibrillary tangles.^8^, ^9^ While the cause of AD has not been confirmed, the well‐known “amyloid hypothesis” theorizes that these Aβ aggregates play the primary role in the pathogenesis and progression of AD.^10^, ^11^ However, there is growing concern that the field should move beyond the “amyloid hypothesis” and shift focus onto other potential pathogenesis hypotheses.^12^ Still, the “amyloid hypothesis” is not yet ruled out, with potential clinical failures attributed to administering therapies too late in AD development to elicit any therapeutic effect.^13^ For example, the A4 study coordinated by the University of Southern California's Alzheimer's Therapeutic Research Institute seeks to preemptively treat older individuals at risk of AD with Aβ aggregate‐reducing therapies to determine whether proactive treatment prevents AD development.^14^ While AD is extensively researched, much remains to be discovered about this clinically devastating and biologically complex disease.

**FIGURE 1:**
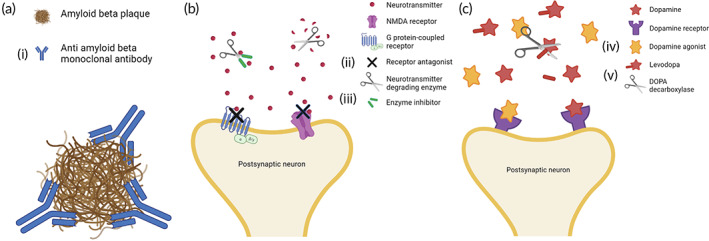
Schematic showing therapy mechanisms utilized by approved AD and PD drugs. a, b, and c, respectively, show therapy mechanisms specific to AD, common overlapping therapy mechanisms, and therapy mechanisms specific to PD. AD, Alzheimer's disease; PD, Parkinson's disease

Figure 1 displays therapy mechanisms utilized by currently FDA‐approved therapeutics for AD and PD and Table 1 lists the specific approved therapies. Several therapies have been approved by the FDA to relieve the symptoms, but not progression of AD. The main limitation of these symptomatic therapies is their inability to slow, stop, or reverses the progression of the disease. These therapies include the acetylcholinesterase inhibitors galantamine hydrobromide (Razadyne, approved in 2001), donepezil hydrochloride (Aricept, approved in 2006), and rivastigmine tartrate (Exelon, approved in 2007), and previously tacrine hydrochloride (Cognex, approved in 1993), which was withdrawn in 2013 due to hepatotoxicity.^15^ These therapies manage symptoms of dementia and mental decline via preserving acetylcholine (ACh) levels in the synaptic gaps between neurons by preventing its degradation by acetylcholinesterase.^15^ Memantine hydrochloride (Namenda, approved in 2003) is another FDA‐approved symptomatic treatment for AD.^16^ Memantine hydrochloride is an N‐methyl‐d‐aspartate receptor (NMDAR) antagonist that aims to prevent excess glutamate from overstimulating neurons and causing damage from excitotoxicity.^16^ Suvorexant (Belsomra), originally a dual orexin receptor antagonist used for insomnia, was approved in 2020 to treat sleep disorders in AD.^17^

**TABLE 1:** All FDA‐approved therapies for AD and PD

No.	Name	Disease	Therapy Mechanism	Approval Year	Notes
1	Razadyne (Johnson & Johnson)	AD	Neurotransmitter system (iii)	2001	Acetylcholinesterase inhibitor^15^
2	Namenda (Abbvie)	AD	Anti‐excitotoxicity (ii)	2003	NMDA receptor antagonist^16^
3	Aricept (Eisai/Pfizer)	AD	Neurotransmitter system (iii)	2006	Acetylcholinesterase inhibitor^15^
4	Exelon (Novartis)	AD	Neurotransmitter system (iii)	2007	Acetylcholinesterase inhibitor^15^
5	Belsomra (Merck)	AD	Neurotransmitter system (ii)	2020	Dual orexin receptor antagonist^17^
6	Aduhelm (Biogen)	AD	Anti‐aggregation (i)	2021	Anti‐amyloid beta monoclonal antibody^18^
7	Cogentin (Merck)	PD	Neurotransmitter system (ii)	1954	Anticholinergic^23^
8	Eldepryl (Somerset)	PD	Neurotransmitter system (iii)	1988	Monoamine oxidase inhibitor^23^
9	Mirapex (Pharmacia)	PD	Neurotransmitter system (iv)	1997	Dopamine agonist^24^
10	Requip (GlaxoSmithKline)	PD	Neurotransmitter system (iv)	1997	Dopamine agonist^23^
11	Tasmar (Hoffman‐La Roche, Inc)	PD	Neurotransmitter system	1998	COMT inhibitor^23^
12	Comtan (Novartis)	PD	Neurotransmitter system	1999	COMT inhibitor^23^
13	Stalevo (Novartis)	PD	Neurotransmitter system (v)	2003	Combination of carbidopa, levodopa, and COMT inhibitor^23^
14	Symmetrel (Endo Pharmaceuticals)	PD	Anti‐excitotoxicity (ii)	2003	NMDA glutamate receptor antagonist^23^
15	Artane (Wyeth‐Ayers Research)	PD	Neurotransmitter system (ii)	2003	Anticholinergic^23^
16	Parcopa (Schwarz Pharma)	PD	Neurotransmitter system (v)	2004	Combination of carbidopa and levodopa^25^
17	Apokyn (Brituswip)	PD	Neurotransmitter system (iv)	2004	Dopamine agonist^23^
18	Zelapar (Valeant Pharmaceuticals)	PD	Neurotransmitter system (ii)	2006	Monoamine oxidase inhibitor^23^
19	Azilect (Teva Pharmaceuticals)	PD	Neurotransmitter system (ii)	2006	Monoamine oxidase inhibitor^23^
20	Neupro (Schwarz BioSciences, Inc)	PD	Neurotransmitter system (iv)	2006	Dopamine agonist^26^
21	Requip XL (GlaxoSmithKline)	PD	Neurotransmitter system (iv)	2008	Dopamine agonist^23^
22	Mirapex ER (Boehringer Ingelheim)	PD	Neurotransmitter system (iv)	2010	Dopamine agonist^24^
23	Sinemet (Merck)	PD	Neurotransmitter system (v)	2014	Combination of carbidopa and levodopa^27^
24	Sinemet CR (Merck)	PD	Neurotransmitter system (v)	2014	Combination of carbidopa and levodopa^27^
25	Rytary (Impax)	PD	Neurotransmitter system (v)	2015	Combination of carbidopa and levodopa^23^
26	Duopa (AbbVie)	PD	Neurotransmitter system (v)	2015	Combination of carbidopa, levodopa, and COMT inhibitor^23^
27	Xadago (Newron Pharmaceuticals)	PD	Neurotransmitter system (ii)	2017	Monoamine oxidase inhibitor^28^
28	Gocovri (Adamas Pharmaceuticals)	PD	Anti‐excitotoxicity (ii)	2017	NMDA glutamate receptor antagonist^29^
29	Inbrija (Acorda Therapautics)	PD	Neurotransmitter system (v)	2018	Inhaled levodopa^30^
30	Osmolex (Osmotica)	PD	Anti‐excitotoxicity (ii)	2018	NMDA glutamate receptor antagonist^31^
31	Nourianz (Kyowa Kirin, Inc)	PD	Neurotransmitter system (ii)	2019	Adenosine A2A receptor antagonist^32^
32	Ongentys (Neurocrine Biosciences)	PD	Neurotransmitter system	2020	COMT inhibitor^33^
33	Kynmobi (Sunovion)	PD	Neurotransmitter system (iv)	2020	Dopamine agonist^34^

*Note*: The roman numerals in parentheses refer to mechanisms illustrated in Figure 1. COMT inhibitors and carbidopa act systemically and hence are not represented in the above schematic.

Abbreviations: AD, Alzheimer's disease; COMT, catechol‐O‐methyl‐transferase; FDA, US Food and Drug Administration; NMDA, N‐methyl‐d‐aspartate; PD, Parkinson's disease.

In 2021, Aducanumab (Aduhelm), an anti‐Aβ monoclonal antibody, became the first Aβ plaque‐reducing therapy for AD to be granted FDA approval.^18^ However, the efficacy of aducanumab is highly controversial since phase III clinical trials were inconclusive in demonstrating clinical benefits, namely improvements in cognitive function and reduction of dementia.^18^ Patients in one study experienced a reduction in AD‐related cognitive decline while those in another study did not experience a statistically significant reduction. Clinical limitations of aducanumab include the several treatment‐emergent adverse events (TEAEs) reported during early phase clinical trials investigating its safety.^19^ Totally, 41.3% of patients in the EMERGE and ENGAGE clinical trials experienced amyloid‐related imaging abnormalities (ARIA), the most common type of TEAE observed, including brain edema, sulcal effusion, and hemosiderin deposits from brain hemorrhage, and 26% of these patients experienced associated symptoms.^20^ All cases of symptomatic ARIA were reported as serious adverse effects. However, no patients were hospitalized due to ARIA, and most ARIA cases resolved within 4–12 weeks.^21^ The FDA granted accelerated approval of aducanumab due to its consistent success with reducing Aβ levels in AD patients, suggesting a “reasonable likelihood” for clinical improvement, while requiring phase IV clinical trials to prove clinical benefit by February of 2030.^22^ If this deadline is not met, FDA approval for aducanumab will be withdrawn. While the clinical benefit of aducanumab has not yet been verified, its availability on the market is a major milestone for AD therapeutic development as it is the first available drug with the potential to slow the progression of AD. Despite these advancements, the existing therapies available for the treatment of AD, while offering significant improvement in management of AD symptoms, are either disputable in efficacy or restricted to providing solely symptomatic relief. AD patients are yet to gain access to a fully approved drug that can reliably slow or stop the progression of AD. By analyzing data from completed and ongoing AD clinical trials, this study will enable researchers to comprehend the current clinical frontier for AD therapeutic development.

### 2.2 Parkinson's disease

PD is the second most common neurodegenerative disorder with characteristic motor symptoms including tremor, rigidity, bradykinesia, and postural instability. PD patients may also experience nonmotor symptoms including sleep disturbances, psychotic symptoms, sensory disorders, mood disturbances, and cognitive impairment. PD occurs after the loss of dopaminergic neurons in the pars compacta of the substantia nigra, associated with the presence of intracellular aggregates of misfolded alpha‐synuclein.^35^, ^36^ The exact cause of PD remains unknown, but several implicated processes include neuroinflammation,^37^, ^38^ mitochondrial dysfunction,^39^, ^40^, ^41^ oxidative stress,^42^ and defective protein homeostasis.^43^, ^44^

Table 1 shows a list of key approved therapies for PD. The most prominent approved treatments for the motor symptoms of PD are medications containing levodopa and carbidopa, such as Sinemet, Paracopa, Rytary, and Duopa. Levodopa is a dopamine precursor,^45^ and carbidopa is a dopa‐decarboxylase inhibitor that reduces extracerebral metabolism of levodopa before it has crossed the protective blood‐brain barrier (BBB),^46^ enhancing levodopa brain bioavailability. Another class of FDA‐approved treatments is catechol‐o‐methyl‐transferase (COMT) inhibitors (such as Comtan, Tasmar, Ogentys), which have achieved success when used in conjunction with carbidopa/levodopa medication. These drugs function by inhibiting the enzyme COMT, thus preventing extracerebral degradation of levodopa and increasing levodopa plasma concentration.^47^ Dopamine agonists can cross the BBB and mimic the effect of dopamine by binding to dopamine receptors,^48^ reducing dyskinesia. Carbidopa and COMT inhibitors reduce the required dosage of levodopa. The current FDA‐approved drugs that act as dopamine agonists are Mirapex, Requip, Apokyn, Kynmobi, and Neupro. Monoamine Oxidase Type B (MAO‐B) is an enzyme that breaks down dopamine in the brain.^49^ MAO‐B inhibitors such as Eldepryl, Zelapar, Azilect, and Xadago have succeeded in gaining FDA approval. NMDA glutamate receptor antagonists including Symmetrel, Gocovri, and Osmolex have been approved by the FDA to treat dyskinesia. Adenosine 2A antagonists such as Nourianz show neuroprotective effects in PD patients. Anticholinergic drugs such as Artane and Cogentin have been approved for their ability to relieve tremors by correcting the imbalance between ACh and dopamine in PD patients. It is worth noting that all the approved therapies are symptomatic therapies.

## 3 CLINICAL TRIAL DATA SET ORGANIZATION

**FIGURE 2:**
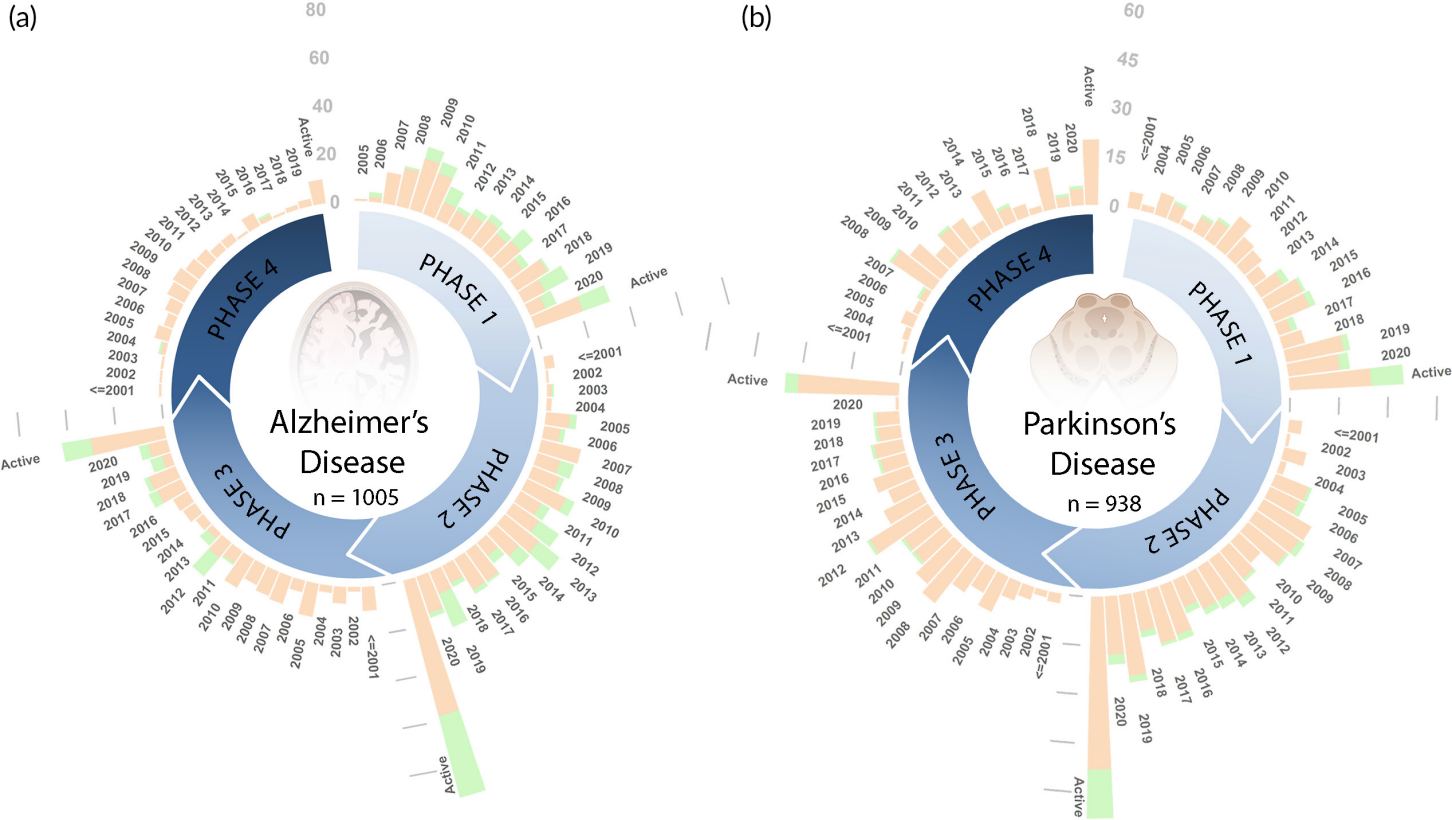
All AD and PD interventional trials fitting the criteria detailed in Section 3. One thousand and five AD trials and 938 PD trials are plotted as a function of the clinical phase and completion year. In the stacked bars, orange color indicates “Drug” and green color indicates “Biologic” therapies. “Biologic” therapies are becoming more popular and are increasingly more successful through the clinical phases. AD, Alzheimer's disease; PD, Parkinson's disease

All clinical trials analyzed were originally sourced from data available from the clinicaltrials.gov database. We eliminated “observational” trials to focus our analysis on “interventional” trials. Within “interventional” trials, we further eliminated trials that were device‐, diagnostic, surgical procedure‐, implant‐, behavioral‐, and radiation‐based, concentrating on therapeutic interventions namely drug‐ and biologic‐based therapies. Starting with 2603 AD and 2933 PD clinical trials, we reduced the data set to 1005 AD and 938 PD clinical trials after the aforementioned eliminations. Among these, 57 AD and 40 PD did not report the trial phase, and hence were not included in analyses that broke up trials across phases. Following the convention of clinicaltrials.gov, we grouped therapy types into either “Drug” or “Biologic” with minor adjustments for consistency with the FDA definition of biologics. In addition to cell‐ and protein‐based systems, we allocated antibodies, peptides, proteins, natural products, and microbial products under the “Biologic” classification. Natural products refer to plant extracts that contain a cocktail of molecules, such as ginkgo biloba extract. However, if a single molecule was isolated from a natural source, such as resveratrol or curcumin, we classified it as a small molecule and therefore under the “Drug” classification. Figure 2 displays the total number of clinical trials in each clinical phase as a function of trial completion years and therapeutic type (“Drug” and “Biologic”) for AD and PD since 2000, with “Biologic” trials stacked in green. Active investigations include all clinical trials that were active as of January 2021, when the clinical trial date of completion data set was analyzed.

## 4 ALZHEIMER'S DISEASE THERAPIES IN THE CLINIC

**FIGURE 3:**
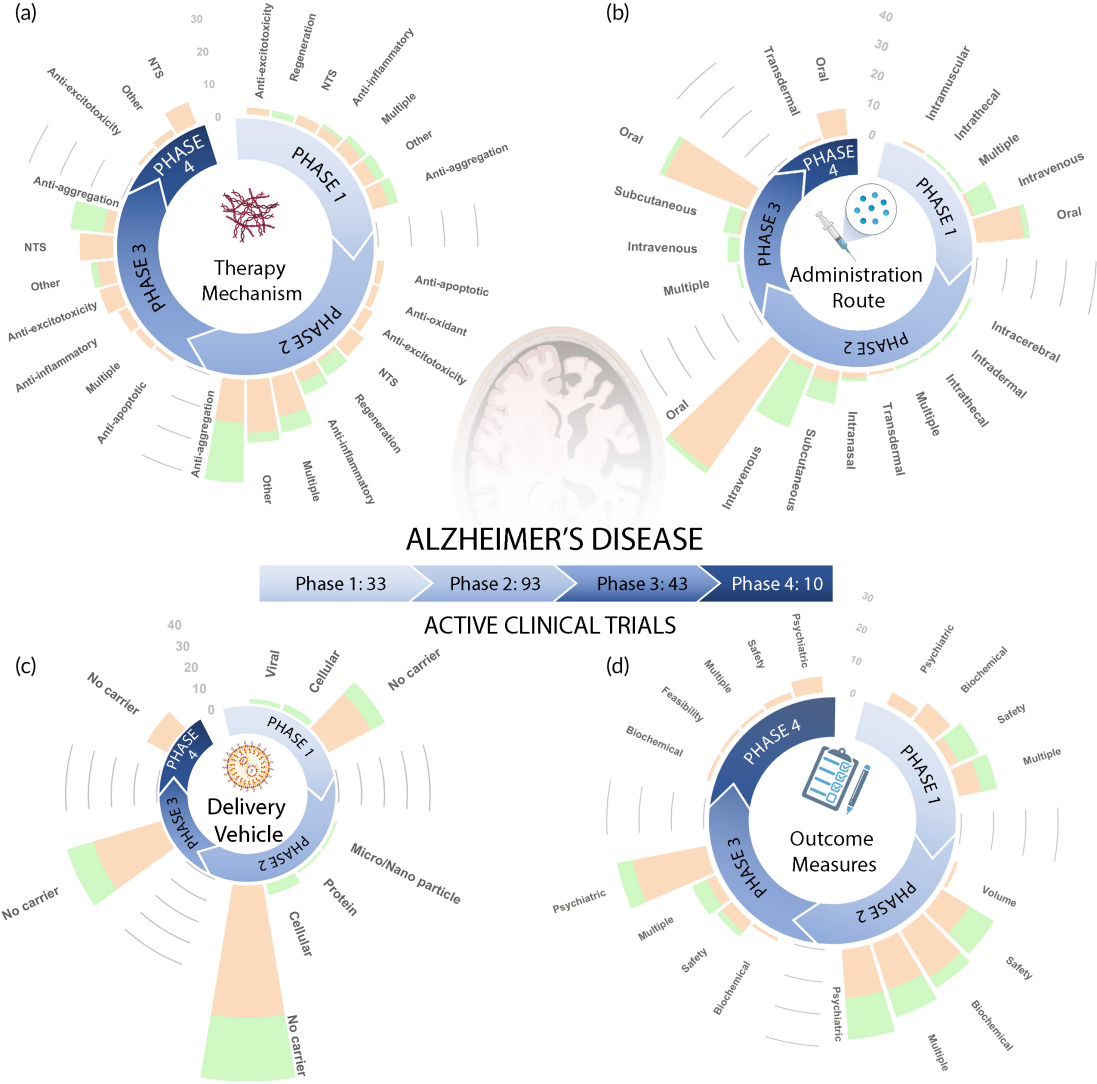
Summary of active AD clinical trials. (a) Therapy mechanism, (b) administration route, (c) delivery vehicle, and (d) outcome measures utilized by 179 active AD trials are plotted as a function of clinical phase. In the stacked bars, orange color indicates “Drug” and green color indicates “Biologic” therapies. The definition of key terms is available in Table A1. Note that liposomes‐based delivery vehicles are binned under micro/nanoparticle category in this article. AD, Alzheimer's disease

**FIGURE 4:**
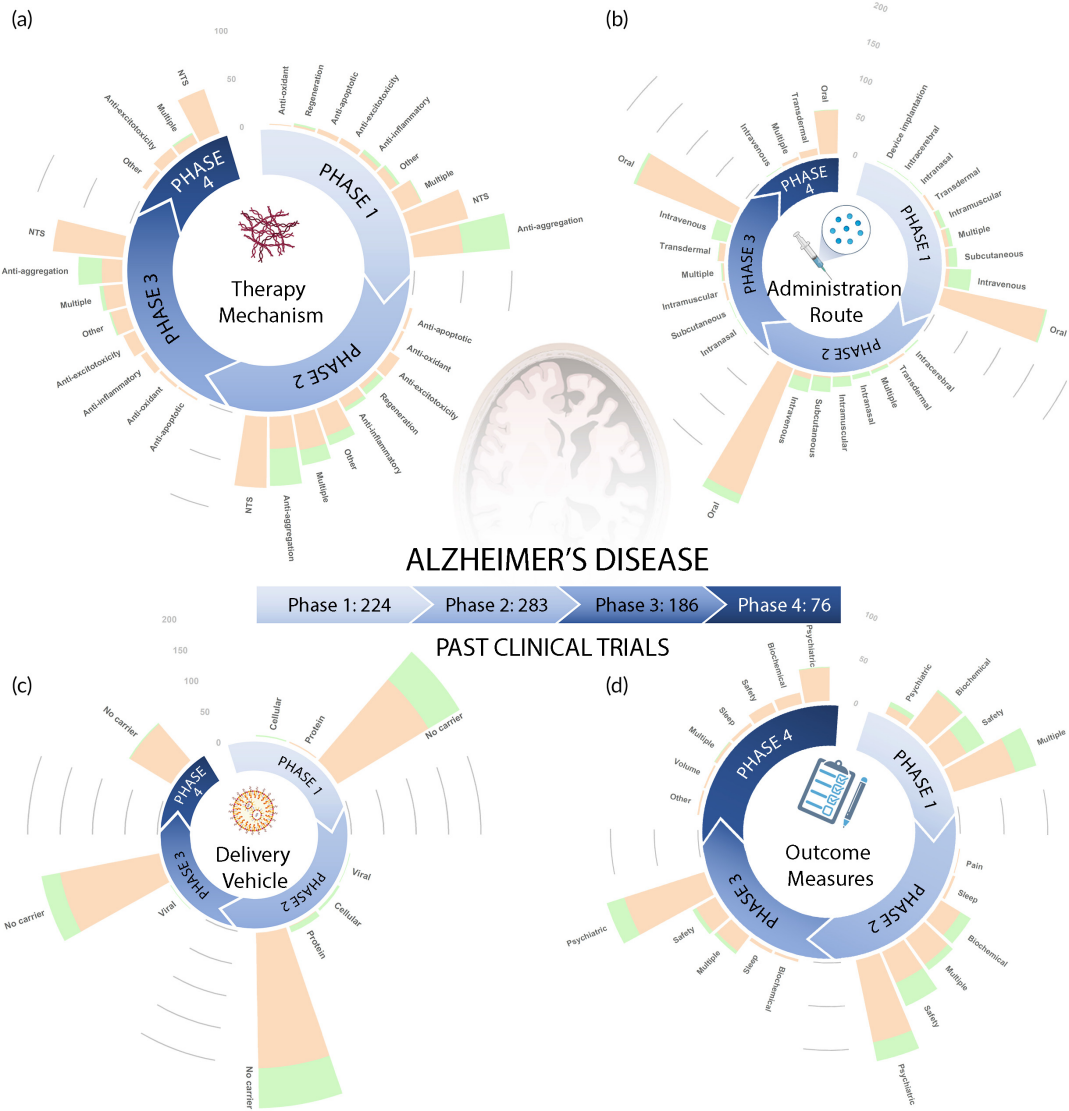
Summary of past AD clinical trials. (a) Therapy mechanism, (b) administration route, (c) delivery vehicle, and (d) outcome measures utilized by 769 past AD trials are plotted as a function of clinical phase. In the stacked bars, orange color indicates “Drug” and green color indicates “Biologic” therapies. The definition of key terms is available in Table A1. AD, Alzheimer's disease

Figure 3 and Figure 4 show a total of 948 clinical trials that are respectively dissected into active (179 trials) and past (769 trials). Note as mentioned in Figure 3, 57 trials (out of 1005) were excluded from the analysis presented in this section due to unreported clinical phase. Considering all the clinical trials represented in figures, we find that there are more trials in the early phases 1 (27% of trials) and 2 (40% of trials), involving basic safety and preliminary efficacy testing, than the later phases 3 (24% of trials) and 4 (9% of trials). Small molecule drug trials make up the majority of clinical trials (76%) in all phases, but account for an even greater proportion of clinical trials (87%) in phases III and IV than biologic therapies. Biologic therapies for AD are relatively newer and more experimental than small molecule therapies for AD and therefore have a relatively smaller number of completed and ongoing clinical trials. However, biologic therapies are increasing in popularity, with biologics accounting for 33% of active trials versus 21% of past trials. Furthermore, several AD clinical trials consist of variations of the currently FDA‐approved small molecule therapies, namely galantamine hydrobromide, donepezil hydrochloride, and rivastigmine tartrate, and are being tested for efficacy across varied patient populations or in combination with other therapies. Table 2 presents 10 currently active clinical trials for AD utilizing a broad range of therapeutic strategies to give an idea of the wide variety of therapeutics under investigation. In the rest of this section we analyze the clinical landscape focusing on the therapy mechanisms, drug administration routes, drug delivery vehicles, and outcome measures as a function of clinical phase and therapy type.

**TABLE 2:** Examples of active AD clinical trials that represent the diverse therapy approaches pursued currently

Name	Phase	Therapy Type	Therapy Mechanism	Administration Route	Delivery Vehicle	Outcome Measures	Trial Number	Notes
LX1001 (Lexeo Therapeutics)	1	Biologic: Gene therapy	Other	Intrathecal	Viral	Safety, biochemical, psychiatric	NCT03634007	Adeno‐associated virus delivery of human apolipoprotein E2 (APOE2) cDNA^50^
Canakinumab/Ilaris (Novartis)	2	Biologic: Antibody	Anti‐inflammatory	Subcutaneous	No carrier	Psychiatric, pharmacokinetics	NCT04795466	Human anti‐IL‐1β monoclonal antibody^51^
MSC‐Exos (Cell Biomedicine Group)	2	Biologic: Cell therapy	Regeneration	Intranasal	Cellular	Safety, psychiatric	NCT04388982	Allogenic adipose mesenchymal stem cell exosomes^52^
ACI‐35.030 & JACI‐35.054 (AC Immune SA)	2	Biologic: Protein/peptide	Anti‐aggregation	Intravenous	Micro/nano particle	Safety, biochemical, psychiatric	NCT04445831	Anti‐phosphorylated tau vaccine, peptide loaded into liposome^53^
ABvac40 (Araclon Biotech)	2	Biologic: Protein/peptide	Anti‐aggregation	Subcutaneous	Protein	Safety, biochemical	NCT03461276	Peptide repeat conjugated to keyhole limpet cyanine (KHL) carrier protein, active vaccine targeting the Aβ40 peptide C‐terminal^54^
CT1812/Elayta (Cognition Therapeutics)	2	Drug: Small molecule	Anti‐aggregation	Oral	No carrier	Biochemical	NCT04735536	Small molecule antagonist of the sigma2 receptor that reduces the affinity of oligomeric Aβ for its receptor^55^
Levetiracetam/Keppra (UCB Pharmaceuticals)	2	Drug: Small molecule	Anti‐excitotoxicity	Oral	No carrier	Psychiatric, EEG	NCT03875638	Binds to synaptic vesicle protein SV2A to reduce neurotransmitter vesicle release rate^56^, ^57^
AC‐1204 Caprylic triglyceride/Tricaprilin (Cerecin)	3	Drug: Small molecule	Anti‐apoptotic	Oral	No carrier	Safety, psychiatric	NCT04187547	Induce mild chronic ketosis to improve mitochondrial metabolism^58^
AVP‐786/Deudextromethorphan Hydrobromide‐Quinidine (Avanir Pharmaceuticals)	3	Drug: Small molecule	Anti‐excitotoxicity	Oral	No carrier	Safety, psychiatric	NCT02446132	Dextromethorphan is a weak antagonist of NMDA receptors and an agonist of sigma 1 receptors; quinidine increases bioavailability^59^
NE3107 (BioVie)	3	Drug: Small molecule	Anti‐inflammatory	Oral	No carrier	Psychiatric, biochemical	NCT04669028	BBB permeable insulin sensitizer that binds ERK, to inhibit ERK and NFKB inflammation^60^

Abbreviations: AD, Alzheimer's disease; BBB, blood‐brain barrier; NMDA, N‐methyl‐d‐aspartate.

### 4.1 Therapy mechanisms

Figures 3a and 4a respectively show the therapeutic mechanisms leveraged by active and past trials. There are seven main therapy mechanisms leveraged by AD trials, namely anti‐aggregation, neurotransmitter system (NTS), anti‐inflammatory, anti‐oxidant, anti‐excitotoxicity, anti‐apoptotic, and regeneration. While many neurological disease processes are intertwined, such as inflammation, oxidative stress, and excitotoxicity,^61^ we categorized therapy mechanism based on their primary intervention target.

Therapies targeting NTS dominate the overall clinical landscape, accounting for 34% of past and 14% of active trials. For NTS intervention, AD therapies mainly targeted the cholinergic system (211), with other therapies targeting the dopaminergic (30), serotonergic (71), GABAergic (7), and norepinephrinergic (5) systems. Cholinergic NTS trials refer to therapies that affect the neurotransmitter ACh. ACh is vital to neuronal signaling involved in several cognitive functions including learning, short‐term memory, and attention.^62^ Drug therapies targeting the cholinergic system typically aim to increase ACh levels, which are lowered in AD. This leads to some symptomatic relief from the cognitive symptoms of AD. These therapies include acetylcholinesterase inhibitors which stop the enzyme‐mediated breakdown of ACh in the synaptic gaps between neurons, nicotinic acetylcholine receptor α7 agonists, and several other medications designed to increase ACh levels.^62^ Also, a large proportion of therapies target the neurotransmitters serotonin and dopamine which are connected to several psychological symptoms of AD including apathy and depression.^63^ These other NTS therapies are also aimed towards providing symptomatic relief rather than stopping or slowing the progression of AD.

Anti‐aggregation therapies dominate the active clinical trial landscape accounting for 30% of active trials increasing from just 14% of past trials. Anti‐aggregation therapies utilize a variety of mechanisms to stop the production, accumulation, and toxicity of hyperphosphorylated neurofibrillary tau tangles and Aβ plaques.^64^ These therapies typically aim to increase clearance by targeting Aβ or tau with monoclonal antibodies such as aducanumab, or reduce Aβ production with β‐secretase 1 inhibitors such as Verubecestat and Lanabecestat,^65^ or γ‐secretase inhibitors such as Begacestat,^66^ to prevent Aβ plaque formation. A greater proportion of clinical trials target Aβ rather than tau, but the proportion of clinical trials targeting tau is greater for “Drug” trials than “Biologic” trials.

A small percentage (7%) of “Biologic” trials investigate regeneration therapies, mostly consisting of mesenchymal stem cell (MSC) transfusion for promoting neuronal growth and regeneration. We also included the category Multiple for clinical trial therapies that intervened via more than one of the seven approaches. Pleiotropic drugs fall in the category of Multiple as these therapeutics often exert multiple effects in concert, such as ginkgo biloba which has anti‐aggregation, anti‐oxidant, anti‐inflammatory, and cholinergic modulation properties.^67^, ^68^ The therapy mechanism Other encompasses all other therapeutic approaches that do not fit within the seven outlined categories. Other therapy mechanisms include drugs that modulate the microbiome, hormonal systems, neurovascular system, or glucose metabolism.

Anti‐aggregation approaches have been commonly used in past clinical trials, and have increased in popularity among currently active trials. Anti‐aggregation was the most common approach in phase 2, and second most common approach in phase 1 and 3 clinical trials in the past, and is currently the most common therapy mechanism in phase 1 and 2 trials. NTS therapies heavily dominated past clinical trials as the most common approach in phases 1, 2, and 4, but have considerably decreased in numbers among currently active trials, especially for newer phase 1 and 2 investigations. Overall, NTS therapies previously and anti‐aggregation therapies presently dominate the majority of clinical trials, with a lower percentage of therapies targeting one or more of the following disease mechanisms: apoptosis, excitotoxicity, inflammation, and oxidative stress.

In AD animal models, Aβ has been shown to induce apoptosis of neurons.^69^, ^70^ However, its pro‐apoptotic role has not been clearly proven in human studies, and its precise molecular mechanism in promoting apoptosis remains unclear.^71^ Additional research on the specific role of the disialoganglioside GD3, a potential target of anti‐apoptotic therapies, is also needed in order to improve therapeutic strategy.^72^ Several anti‐apoptotic therapies have shown positive results in early clinical trials, but further investigation is needed to confirm these results.^71^

Anti‐excitotoxic therapies target NMDARs and their ligand, the excitatory neurotransmitter glutamate. Excitotoxicity occurs when NMDARs are overstimulated, allowing excess sodium and calcium ions to enter the neuron, generating excessive levels of reactive oxygen species (ROS), exhausting ATP stores in attempt to reestablish ionic equilibrium, and ultimately inducing necrosis or apoptosis.^73^, ^74^ Many anti‐excitotoxic therapies are unable to pass the early stages of clinical trials due to the adverse effects caused by a widespread blocking of NMDAR activity which is essential to normal neuronal function.^75^ Successful anti‐excitotoxic therapies should block NMDAR overstimulation at injured brain regions without impeding essential NMDAR functioning.^76^ For this reason, competitive antagonists of glutamate and glycine are prone to causing TEAEs and are not viable options for AD patients. Some noncompetitive antagonists such as MK‐801 act allosterically to block the ion channel, but blockage for an extended period also leads to TEAEs.^77^ However, antagonists that must first be activated by an agonist before allosteric binding to NMDARs only block NMDAR channels in conditions of excitotoxicity thus providing clinical benefit without TEAEs. One such antagonist is memantine hydrochloride (Namenda), which was approved by the FDA in 2003.^78^

Chronic inflammation can damage brain tissue in AD patients due to excess release of cytokines from overactive microglia, astrocytes, and invading peripheral leukocytes.^79^ Aβ activates the complement cascade, releasing anaphylatoxins, increasing levels of amyloid precursor protein (APP), and producing free radicals.^80^ Clinical trials investigating anti‐inflammatory therapies are complicated by the lack of inflammatory biomarker detection methods to monitor the success of these therapies. Studies on inflammatory markers have produced controversial results due to their inconsistent sampling times and patient populations.^81^ Current therapeutic strategies targeting inflammation include reducing the concentration and inhibiting the action of cytokines, and manipulating microglia to phagocytose Aβ plaques.^82^ Studies investigating the preventive effect of non‐steroidal anti‐inflammatory drugs on AD have shown mixed results, with naproxen (Aleve) and celecoxib (Celebrex) appearing to increase AD risk.^83^

Oxidative stress occurs due to the buildup of ROS and contributes to the development of AD. Anti‐oxidant therapies aim to mitigate damage caused by oxidative stress, which is pronounced in brain regions most affected by AD.^84^ Although several substances that scavenge ROS have been investigated in AD animal models, most anti‐oxidant therapies are unable to significantly lower oxidative stress and have overall shown limited success in human patients.^85^ Furthermore, some anti‐oxidant therapies such as vitamin E may increase oxidative stress under various conditions.^86^ When administered in high dose, anti‐oxidant therapies have the potential to disrupt the body's natural anti‐oxidant defense system.^87^ The majority of clinical trials for anti‐oxidant therapies are currently confined to earlier stages and more research is needed to verify clinical benefit for AD patients.^88^

In addition to anti‐aggregation approaches, alternative therapy mechanisms are gaining in prevalence among currently active clinical trials. Most NTS drug therapies for AD aim to provide symptomatic relief, reducing the emotional burden of patients and caregivers affected by severe memory loss, persistent confusion, and other cognitive, behavioral, and psychological symptoms caused by AD. In contrast, clinical trials not targeting NTS more often aim to slow or stop the progression of AD as they target potential root causes of neuronal death in AD, namely inhibition of aggregation, apoptosis, inflammation, excitotoxicity, and oxidative stress. Therapies targeting these alternative disease mechanisms may have potential for success in mitigating AD progression as further research is conducted in these less explored areas.

### 4.2 Administration route

Figures 3b and 4b show the distribution of AD therapies by administration route for active and past clinical trials, respectively. The majority of “Drug” therapies are delivered orally. Because small molecule drugs can bypass gastrointestinal barriers, oral delivery is favored as the least invasive mode of administration with high patient compliance and convenience.^89^ In contrast, “Biologic” therapies are delivered through an injection, most commonly via intravenous (IV), intramuscular, or subcutaneous (SC) administration. Oral delivery dominates past and present clinical trials, but IV and SC delivery has gained in prominence among currently active phase 1 and 2 trials.

### 4.3 Delivery vehicle

Figures 3c and 4c show the distribution of clinical trials by delivery vehicle for active and past clinical trials, respectively. A very limited proportion of clinical trials use drug carriers. While “Biologic” therapies have a slightly more diverse distribution of delivery vehicles than that of “Drug” trials, both heavily underutilize delivery vehicles, with a vast majority of therapies using no carrier. Increased utilization of delivery vehicles offers potential to improve therapeutic efficacy for AD treatment, as elaborated in Section 6.4. Delivery vehicle approaches are increasing in number among currently active phase 1 and 2 trials.

### 4.4 Outcome measures

Figures 3d and 4d show the distribution of clinical trials categorized by the outcome measures utilized by active and past clinical trials, respectively. Outcome measures can be organized into the categories of Safety, Psychiatric, Biochemical, Sleep, Pain, Volume, Other, and Multiple. Volume refers to the percent change of hippocampal or whole brain volume. Most outcome measures categorized as Safety involve the identification of TEAEs. Common Biochemical outcome measures include cerebrospinal fluid or serum measurements of concentrations of various biomarkers, such as Aβ, tau, or inflammatory cytokines. While biochemical outcome measures are often used for preliminary demonstration that an AD therapy is achieving a significant effect by showing target interaction or some disease‐modifying capability, clinical benefit must be proven via psychiatric outcome measures for long‐term FDA approval. Common psychiatric outcome measures for cognition for AD clinical trials include the mini‐mental status examination,^90^ Alzheimer's disease cognitive scale,^91^ neuropsychiatric inventory,^92^ Alzheimer's disease cooperative study‐activities of daily living,^93^ and changes from baseline cognitive performance in working memory, processing speed, and learning abilities. These methods assess the patients' clinical improvement, or lack thereof, in response to each AD therapy. Psychiatric assessments are the primary outcome measures for both past and present trials for both “Drug” and “Biologic” therapies. Safety and then Biochemical are the next most common outcomes assessed. Many currently active phase 2 and 3 trials observe Multiple outcome measures, mainly composed of Psychiatric, Safety, and Biochemical outcomes.

## 5 PARKINSON'S THERAPIES IN THE CLINIC

**FIGURE 5:**
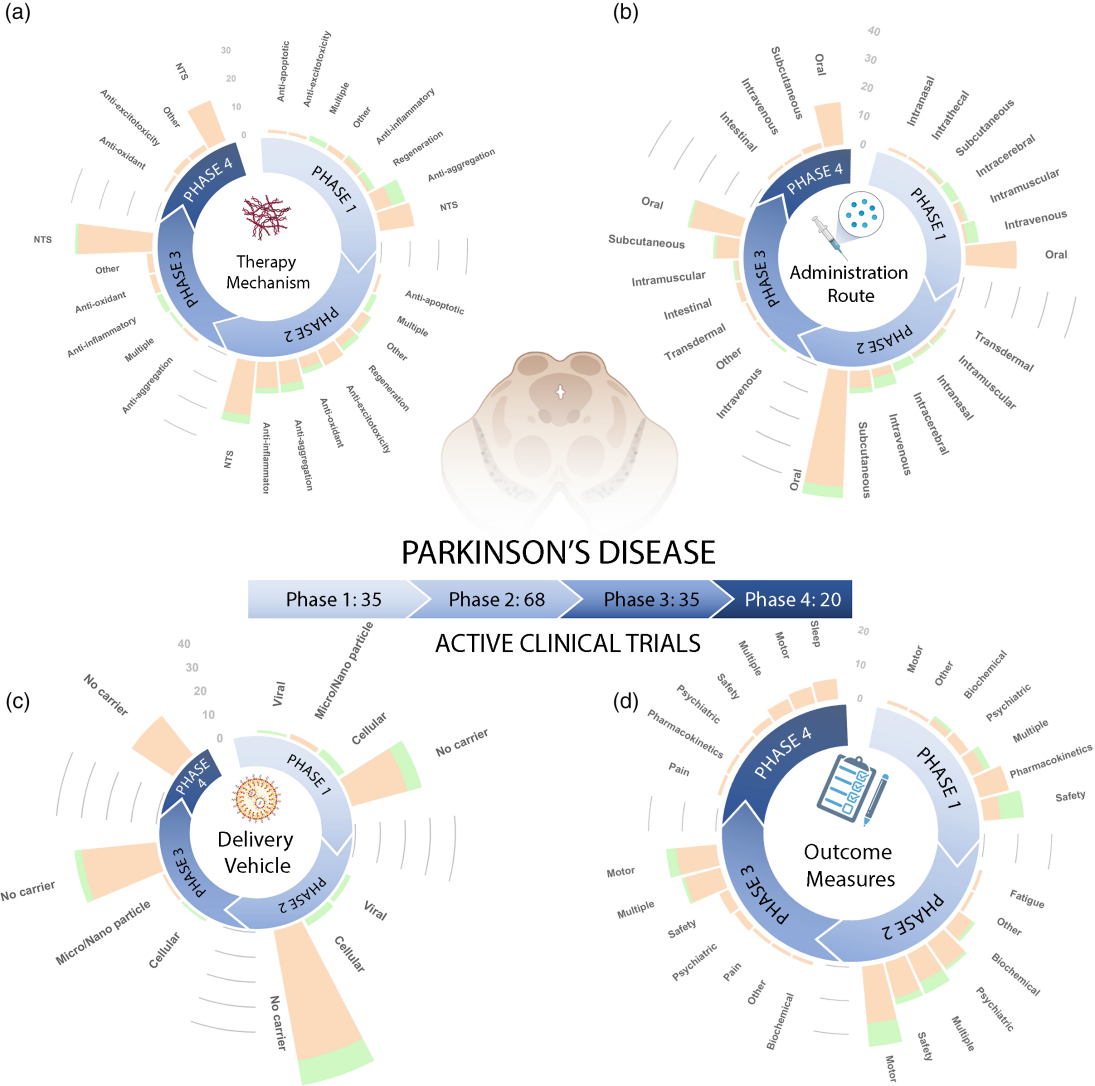
Summary of active PD clinical trials. (a) Therapy mechanism, (b) administration route, (c) delivery vehicle, and (d) outcome measures utilized by 158 active PD trials are plotted as a function of clinical phase. In the stacked bars, orange color indicates “Drug” and green color indicates “Biologic” therapies. The definition of key terms is available in Table A1

**FIGURE 6:**
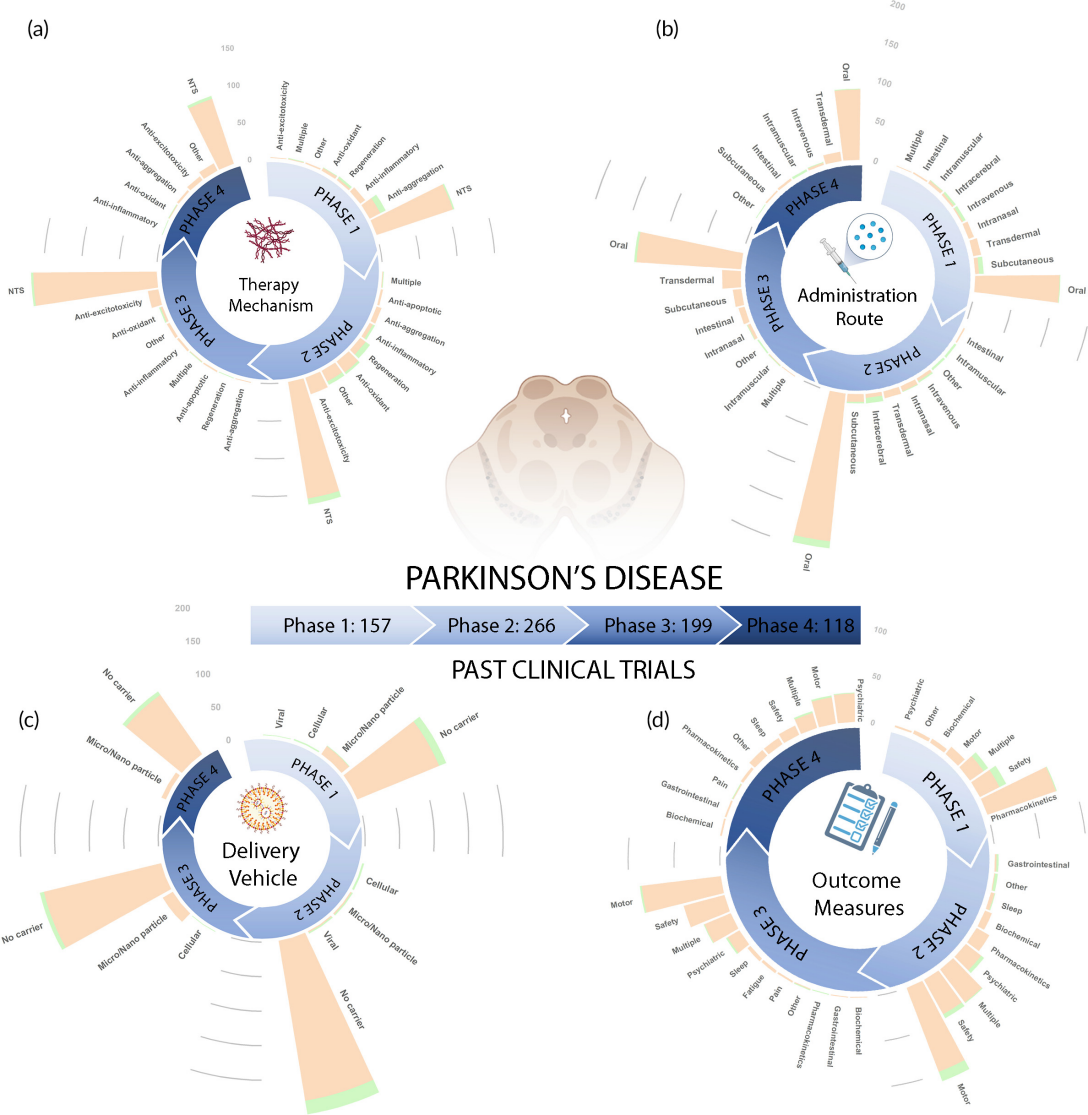
Summary of past PD clinical trials. (a) Therapy mechanism, (b) administration route, (c) delivery vehicle, and (d) outcome measures utilized by 740 past PD trials are plotted as a function of clinical phase. In the stacked bars, orange color indicates “Drug” and green color indicates “Biologic” therapies. The definition of key terms is available in Table A1

Figure 5 and Figure 6 show a total of 898 PD clinical trials that are respectively dissected into active (158 trials) and past (740 trials). Note as mentioned in Figure 3, 40 trials (out of 938) were excluded from the analysis presented in this section due to unreported clinical phase. Considering all the clinical trials represented in the figures, we find that the majority of active clinical trials are in phase 2 (44%), followed by phase 3, 1, and 4, which account for 22%, 22%, and 12% of the total active trials, respectively. A similar ordering is observed in the past clinical trials with phase 2, 3, 1, and 4 accounting for 36%, 27%, 21%, and 16%, respectively. Unsurprisingly, small molecule “Drug” therapies account for the lion's share of the therapy type accounting for 66% and 78% of the active and current trials respectively. “Drug” therapies also have a significantly larger proportion of therapies that have advanced to phase 3 and phase 4 clinical trials, indicating that they are nearing FDA approval or have already been approved for general use. “Biologic” therapies are increasingly becoming popular with protein/peptide‐based, cell‐based, and antibody therapies leading the way by accounting for 28%, 24%, and 21%, respectively of active “Biologic” therapies (see Section 6 for more details). The majority of “Biologic” therapies remain in Phase 1 and Phase 2 as they are relatively new, and target less‐explored mechanisms of PD pathology that are mainly still being evaluated for safety. Table 3 presents 10 currently active clinical trials for PD utilizing a broad range of therapeutic strategies to give an idea of the wide variety of therapeutics under investigation. As with AD, in the rest of this section we analyze the clinical landscape focusing on the therapy mechanisms, drug administration routes, drug delivery vehicles, and outcome measures as a function of clinical trial phases and therapy types.

**TABLE 3:** Examples of active PD clinical trials that represent the diverse therapy approaches pursued currently

Name	Phase	Therapy type	Therapy mechanism	Administration route	Delivery vehicle	Outcome measures	Trial number	Notes
AAV2‐GDNF (Brain Neurotherapy Bio, Inc.)	1	Biologic: Gene therapy	Regeneration	Intracerebral	Viral	Safety	NCT04167540	Adeno‐associated virus delivery of glial cell line‐derived neurotrophic factor^94^
UB‐312 (Vaxxinity)	1	Biologic: Protein/peptide	Anti‐aggregation	Subcutaneous	No carrier	Safety	NCT04075318	Peptide‐based vaccine targetting alpha‐synuclein^95^
Prasinezumab (Hoffmann‐La Roche)	2	Biologic: Antibody	Anti‐aggregation	Intravenous	No carrier	Motor, psychiatric	NCT03100149	Humanized IgG1 monoclonal antibody directed against aggregated α‐synuclein^96^
Exenatide (AstraZeneca)	2	Biologic: Protein/peptide	Anti‐inflammatory	Subcutaneous	No carrier	Biochemical	NCT04305002	Analog of the incretin hormone glucagon‐like peptide (GLP‐1), previously FDA approved for T2D^97^
HB‐adMSCs (Hope Biosciences)	2	Biologic: Cell therapy	Multiple	Intravenous	Cellular	Motor, safety, biochemical	NCT04928287	Autologous adipose‐derived mesenchymal stem cells^98^
TAK‐071 (Takeda)	2	Drug: Small molecule	Neurotransmitter system	Oral	No carrier	Motor, pharmacokinetics	NCT04334317	Muscarinic acetylcholine receptor 1 positive allosteric modulator^99^
Rifaximin (UC San Francisco)	2	Drug: Small molecule	Anti‐inflammatory	Oral	No carrier	Other	NCT03575195	Antibiotic targeting gut microbiota^100^
Cannabidiol (University of Colorado)	2	Drug: Small molecule	Anti‐oxidant	Oral	No carrier	Motor	NCT03582137	Extract from the *Cannabis sativa* plant^101^
UCB0599 (UCB Biopharma SRL)	2	Drug: Small molecule	Anti‐aggregation	Oral	No carrier	Motor, psychiatric	NCT04658186	Small‐molecule α‐synuclein aggregation inhibitor^102^
Apomorphine (US WorldMeds LLC)	3	Drug: Small molecule	Neurotransmitter system	Subcutaneous	No carrier	Motor	NCT02339064	Non‐ergoline dopamine D2 agonist indicated to treat hypomobility^103^

Abbreviation: PD, Parkinson's disease.

### 5.1 Therapy mechanisms

Figures 5a and 6a, respectively, show the same therapeutic mechanisms utilized by active and past clinical trials organized into seven main categories, namely Anti‐aggregation, NTS, Anti‐inflammatory, Anti‐oxidant, Anti‐excitotoxicity, Anti‐apoptotic, and Regeneration.

Majority of PD “Drug” therapies (69.5%) target NTS, accounting for 47% of active and 72% of past trials. Among these, the neurotransmitter dopamine is unsurprisingly the most popular with many trials testing varied doses of levodopa/carbidopa, COMT inhibitors, and AADC inhibitors that reduce levodopa breakdown, and therapies that act as dopamine agonists of D2 or D1/D5 receptors. Other targeted neurotransmitters include serotonin that is employed by drugs that combat nonmotor symptoms of PD, particularly reduction in sleep quality.^104^ ACh has been targeted in therapies aimed at treating motor PD symptoms such as dyskinesia as well as nonmotor symptoms such as overactive bladder.^23^ Trials with histamine assess its effect on daytime sleepiness in PD patients. Prodrugs of norepinephrine such as Droxidopa that can cross the protective BBB, are also tested for their effects on motor and nonmotor symptoms of PD.^32^ Therapies targeting metabotropic glutamate include NMDAR blockers that have been used to reduce dyskinesia.^32^ Adenosine a2a receptor agonists are also being explored for their neuroprotective effects and are being used as add‐on therapies to shorten off time for patients on carbidopa/levodopa.^32^, ^105^ Therapies that target the neurotransmitter GABA are also tested for their effect on sleep quality and motor symptoms.

Other therapy mechanisms leveraged by PD clinical trials include anti‐aggregation therapies such as myeloperoxidase inhibitor AZD3241 that are tested for effectiveness against the toxic aggregation of alpha‐synuclein, known as Lewy body formation.^106^ Anti‐apoptotic therapies such as TCH346 are tested for their efficacy in delaying loss of dopaminergic neurons.^107^, ^108^ Anti‐excitotoxicity drugs such as memantine block glutamate receptors are tested for reducing involuntary movements (dyskinesias) in PD.^109^ Anti‐inflammatory therapies such as Omega‐3 fatty acid Docosahexaenoic acid are tested for reducing neuroinflammation and consequent improvement in dyskinesia. Small molecule drugs targeting regeneration use neurotrophic factors to protect and repair neurons. We also included the category Multiple for clinical trial therapies that intervened via more than one of the seven approaches. Pleiotropic therapeutics such as stem cells fall in the category of multiple, as these therapeutics often exert multiple effects in concert such as regeneration, anti‐apoptosis, and neurotransmitter production. The therapy mechanism Other for PD include therapies that modulate the microbiome, opioid receptors, hormones such as through antidiuretics, or metabolism.

Similar to AD, there is an increased diversity in “Biologic” therapy mechanisms in active clinical trials. Anti‐aggregation is a popular mechanism of action employed by several recent “Biologic” therapies such as UB‐312 and Affitope PD01A, which are focused on preventing Lewy body formation in dopaminergic neurons to prevent their degeneration.^105^ Anti‐inflammatory therapies such as Exenatide are currently investigated for their potential to reduce dopaminergic neuron degeneration. Many past and active “Biologic” clinical trials have utilized Regeneration based therapies. Dopaminergic neurogenesis is a key goal of these therapies, with many trials employing stem cells which can differentiate into dopaminergic neurons. A significant proportion of Regeneration therapies also test the utility of neurotrophic factors such as GDNF and neurturin to protect dopaminergic neuron end terminals.^105^ “Biologic” therapies also targeted NTS, with 50% of these targeting dopamine and the remaining 50% targeting ACh.

Overall, there is a clear dominance of NTS therapies across past and active trials. Interestingly, the only exception to this is the active phase 1 trials, which are dominated by Anti‐aggregation therapies. We also see an interesting paradigm shift in the diversity of therapy mechanisms leveraged by “Biologics.” Anti‐aggregation, NTS, and Anti‐inflammatory therapies in phase 1, which were once dominated by small molecule drugs are now completely driven by biologics. The eventual outcome of these promising changes are eagerly awaited.

### 5.2 Administration route

Figure 5b and 6b respectively show the administration routes utilized by active and past trials. Overall, there are no significant changes in the diverse administration routes employed by past and present clinical trials, with oral administration still being the preferred route. The vast majority of drug therapies (78%) are delivered orally in the form of tablets, increasing ease for patients to independently administer the therapy. A notable fraction of small molecules (12%), especially dopamine agonists, are also administered subcutaneously or transdermally via patches and topical creams. “Biologic” therapies are mostly delivered via IV administration, whereas those in the form of natural products are commonly administered orally. A large number of “Biologic” therapies are delivered intracerebrally through direct implantation into the brain. These therapies tend to be stem cell therapies which involve stem cells that are placed via intraputaminal infusions directly into the area of the brain in which they are needed. Neurotrophic factors are also mostly intracerebrally administered.

### 5.3 Delivery vehicle

Figures 5c and 6c, respectively, show the distribution of delivery vehicles utilized by active and past trials. “Drug” therapies are mostly administered freely without a carrier. Extended‐release micro/nano particles are also utilized for the continuous delivery of small molecule drugs such as rotigotine, prolonging the effects of the therapy and providing sustained improvements in symptoms. Compared to “Drug” therapies, “Biologic” therapies leverage more diverse drug delivery vehicles. Many “Biologic” therapies have cellular delivery vehicles, which include therapies utilizing stem cells that will differentiate into dopaminergic neurons. “Biologic” genetic therapies utilize non‐pathogenic viral vectors to transfer healthy copies of genes to the patient. However, very few “biologic” therapies use microparticles or nanoparticles to deliver the therapy, suggesting an area in need of further development. The vast majority of past and active clinical trials across therapy types did not use a delivery vehicle, revealing the untapped potential of using delivery vehicles to optimize efficacy of PD treatment (see Section 6.4 for a more detailed discussion).

### 5.4 Outcome measures

Figures 5d and 6d, respectively, show outcome measure used to determine success of the therapy across active and past trials. Most of the “Biologic” therapies (which tended to be in early clinical trial phases) were being monitored for safety, measuring the occurrence TEAEs, or vital sign abnormalities. “Biologic” therapies in phase 2, mostly NTS and Anti‐aggregation therapies, are dominantly evaluated for changes in motor symptoms. In contrast, the more established “Drug” therapies were being evaluated for improvements in motor and nonmotor symptoms, commonly using Movement Disorders Society‐Unified Parkinson's Disease Rating Scale scores to evaluate efficacy. Other common outcome measures include drug pharmacokinetics, which is usually obtained via plasma drug concentration measurements in patients at various intervals, daytime sleepiness, which is usually evaluated using the Epworth Sleepiness Scale, and pain intensity, which is usually measured using the Visual Analog Scale.^110^ Biochemical outcomes including hematology measures such as basophils to leukocytes ratio, eosinophils to leukocytes ratio, lymphocytes to leukocytes ratio, and reticulocytes to erythrocytes ratio are also utilized in PD trials.

## 6 INTERSECTIONS IN CLINICAL APPROACHES

Despite different etiology of disease for AD and PD, these neurodegenerative diseases have parallel roots of potential causes of disease onset. While AD primarily affects cognitive function and PD primarily affects motor function, there are at times a partial overlap in symptoms across the two neurodegenerative diseases, with interrelated pathologenesis as well. Both AD and PD are afflicted with protein aggregates, whether Aβ, tau,^8^ or α‐synuclein,^35^ dysregulation of NTSs,^63^, ^104^, ^105^ neuronal death,^105^, ^111^ inflammation,^37^, ^112^ oxidative stress,^42^, ^113^ and excitotoxicity.^114^, ^115^ Furthermore, similar therapies utilizing memantine hydrochloride, or cholinergic, dopaminergic, and serotonergic system modulators are explored for both neurodegenerative diseases. As a result, there is a meaningful overlap in the therapeutic approaches and strategies leveraged to treat these diseases (Figure 8a). In the next section, we critically evaluate the success rate of past clinical trials and three emerging aspects relevant to the AD‐PD clinical intersection.

### 6.1 Clinical outcomes based on therapy mechanisms

**FIGURE 7:**
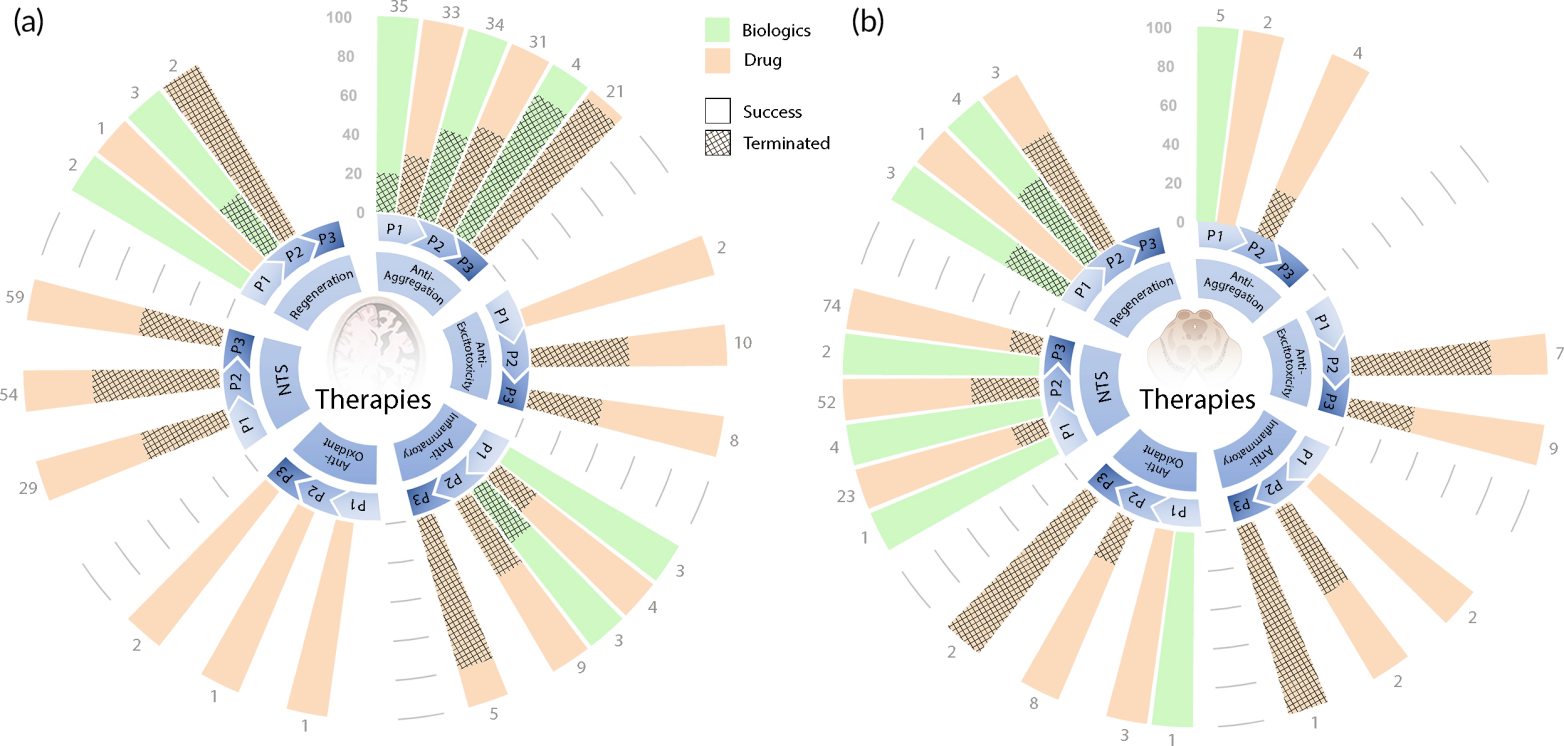
Clinical outcomes based on therapy mechanism for “Drug” and “Biologic” trials. Success and termination percentiles for (a) AD and (b) PD clinical trials. A total of 375 out of 769 AD past trials have reported results (either through clinical trials.gov or via supported publications), whereas 213 out of 740 past PD trials have reported results. The numbers on top of the bars indicate the total number of trials represented by the bar. AD, Alzheimer's disease; PD, Parkinson's disease

Figure 7 displays the percentiles of successful and terminated clinical studies based on therapy mechanism for past “Drug” and “Biologic” clinical trials (2001–2020). A total of 375 out of 769 AD past trials have reported results (either through clinicaltrials.gov or via related publications), whereas 213 out of 740 past PD trials have reported results. NTS “Drug” clinical trial outcomes for AD and PD reveal similarities with the lowest success rate in phase 2 compared to that of phases 1 and 3. This is also expected from broad clinical trial success rates over the last couple of decades.^116^, ^117^, ^118^ However, there are deviations from this expected trend for some other therapy mechanisms for “Drug” and “Biologic” trials. One potential reason for deviations is the limited number of clinical trials with reported results for these other categories. This is especially apparent for categories with 100% success or 100% failure rate, where <5 clinical trials account for the percentile. It is also worth noting a large number of trials also evaluate repurposed FDA‐approved drugs, which often enter the clinical gauntlet at phase 2 or higher. This is the reason for the absence of phase 1 data for PD Anti‐excitotocity category. Nonetheless, this analysis reveals several interesting trends.

Anti‐aggregation clinical studies for AD exhibit a high failure rate of >80% for “Drug” and “Biologic” candidates. Potential explanations include targeting the wrong pathological substrates, administering treatment too late in disease progression, and other issues as discussed by Mehta et al.^119^ Anti‐inflammatory “Drug” candidates for both AD and PD exhibited greater than 80% and 100% failure in phase 3, respectively, which may also possibly be attributed to AD/PD treatment window. The lone successful phase 3 trial of an anti‐inflammatory agent targeting AD is antibiotic Doxycycline, which showed promising results,^120^ but is not yet an approved therapy. The apparent success of anti‐oxidant therapies for AD stem from investigation of vitamin E^121^ or combination of vitamin E and memantine. Anti‐oxidant therapies, however, have not been successful for tackling PD. Furthermore, “Biologic” trials outperformed “Drug” trials in terms of percentile success across all therapy types and mechanisms. This is notable in the case of PD NTS therapies where protein/peptide based “Biologic” therapies (e.g., Rimabotulinumtoxinb) have enjoyed considerable success. While “Drug” candidates have not surpassed the clinical trial barrier for curative treatments, “Biologic” therapies offer potential for achieving neuroprotection beyond symptomatic improvement.

### 6.2 The evolving landscape of therapeutic mechanisms of disease intervention

Both AD and PD clinical trials have exhibited a shift in distribution away from the predominant NTS therapeutic mechanism approaches to a more varied distribution of therapeutic interventions. This trend is most notable and consistent since 2011–2015 until the present. Considering NTS therapies solely manage the symptoms of AD and PD, the growth of clinical trials exploring alternative therapeutic mechanism approaches illustrates researchers' and clinicians' attempts to combat potential root causes of neurodegenerative diseases to inhibit disease progression.

**FIGURE 8:**
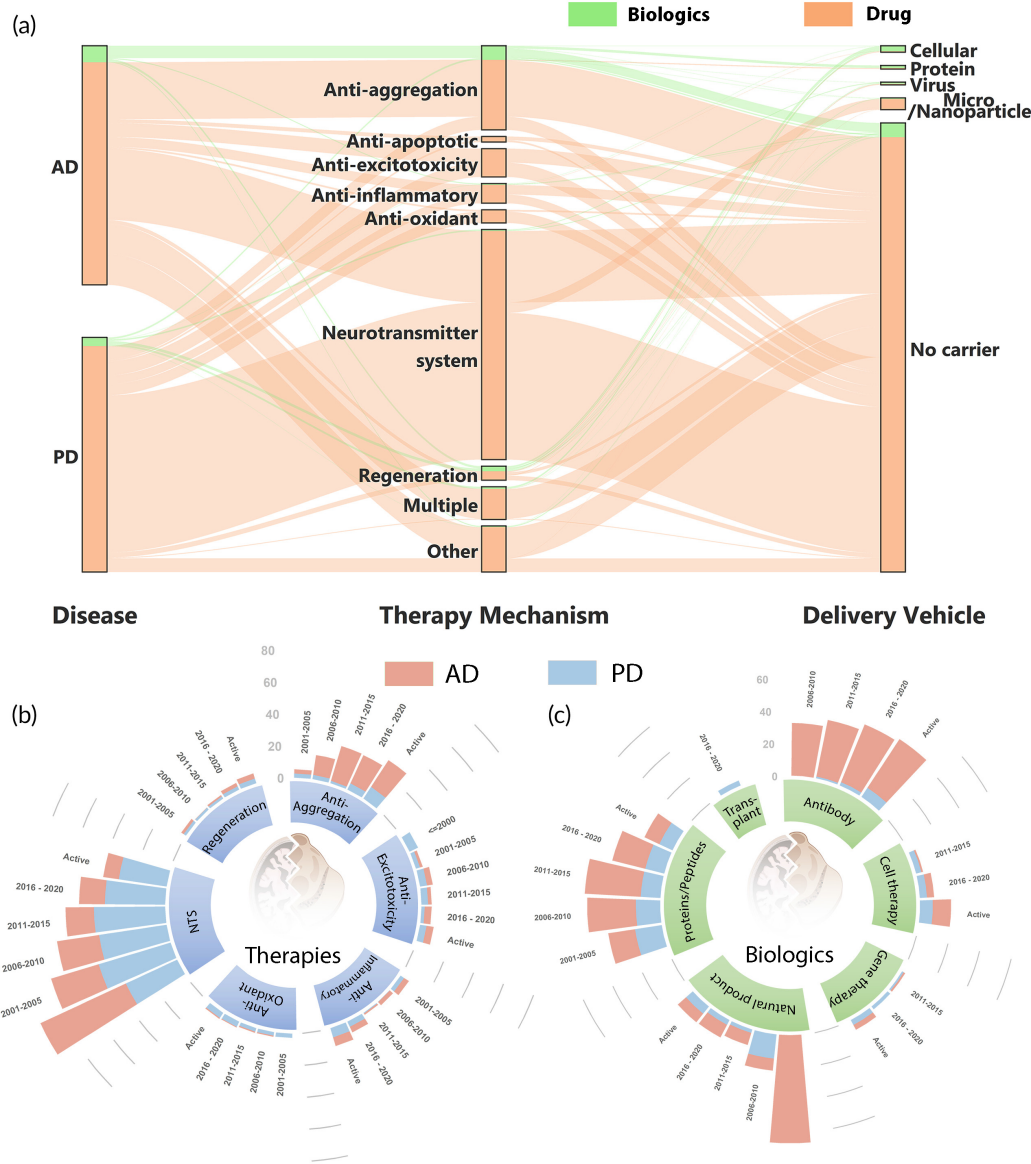
Clinical intersections in the treatment of AD and PD. (a) Alluvial plot showing the intersections in the therapy mechanisms and drug delivery vehicles. Circular bar charts showing the distribution of clinical trials across the six most common (b) therapy mechanisms and (c) “Biologic” therapy types over 5‐year bins, encompassing all trials that were completed or terminated during that period of time. The radial axis denotes percentage of total number of AD and PD trials present in a 5‐year bin. Note that the completion year was used to date the trials. AD, Alzheimer's disease; PD, Parkinson's disease

There has been widespread investment in cholinergic system modulation and anti‐Aβ strategies without fruition of a therapeutic that effectively mitigates AD progression. Altogether, NTS therapies accounted for 33% of past clinical trials for AD, dominating as the most common therapy mechanism in all phases (Figure 4a). Meanwhile, Anti‐aggregation approaches increased from 2001–2005 to 2006–2010, and have since remained relatively constant from 2011 to present (Figure 8b). However, the future is uncertain as to whether Anti‐aggregation approaches will gain or lose popularity as the amyloid hypothesis is being carefully scrutinized and alternative hypotheses are explored.^13^ Reflective of the field's shift in encouraging exploration of alternative therapeutic intervention strategies for AD treatment, there is an expansion of therapy mechanism approaches among currently active clinical trials. The percent of NTS clinical trials has reduced over time, while Regeneration, Anti‐excitotoxicity, and Anti‐inflammatory therapy clinical trials have increased since 2011–2015 (Figure 8b). These trends are further illustrated in Figure 3a, where Anti‐inflammatory and Anti‐excitotoxicity therapies occupy the greatest percentage of clinical trials for phase 1, 2, and 4 clinical trials, respectively. In combination with lack of clinical success of Anti‐aggregation and NTS approaches beyond symptomatic relief, expansion of the fields of cell therapy for anti‐inflammation and regeneration with stem cells,^122^, ^123^ and critical re‐examination of excitotoxicity and targeting NMDARs after the successes of memantine and riluzole^124^ have contributed to the broadening of therapeutic clinical approaches for neurodegenerative diseases. Meanwhile, to a lesser extent, NTS trials for PD have also decreased since 2011–2015. The breadth of therapeutic mechanism approaches combating PD has also expanded as Anti‐aggregation, Anti‐inflammatory, Anti‐oxidant, and Regeneration clinical trials have increased since 2011–2015. Notably, in a shift reminiscent of the AD clinical landscape, the active phase 1 trials of PD are dominated by Anti‐aggregation therapies (Figure 5a).

### 6.3 The evolving distribution of biologic therapeutics

“Biologic” therapies are becoming more prevalent in clinical trials for both AD and PD as evidenced in recent years and especially for currently active phase 1, 2, and 3 clinical trials. Figure 8c displays the percentage of each type of “Biologic” used for AD and PD. The majority of Antibody therapy types are monoclonal antibodies targeting Aβ or tau for AD, and targeting α‐synuclein for PD. For AD, the investigation of antibodies has remained relatively stable over the last 20 years, constituting (~35%) of “Biologic” therapies. On the other hand, Antibody approaches are gaining popularity in PD clinical trials, with percentage of trials doubling from 2011–2015 to 2016–2020, and then tripling from 2016–2020 to currently active clinical trials. Cell therapy and Gene therapy “Biologic” therapies have undergone a similar trend. Cell therapy trials increased from 1% to 4% to 11% for AD, and 3% to 4% to 8% for PD, and Gene therapy trials increased from 1% to 0% to 3% for AD, and 1% to 2% to 3% for PD from 2011–2015 to 2016–2020 to present. Interestingly, Natural product “Biologic” therapies exhibited a sharp decrease in percentage after 2005 for AD and after 2020 for PD clinical trials. Proteins/peptides‐based “Biologic” therapies (note that antibodies are categorized separately) have also gradually decreased in the total percentage of “Biologic” clinical trials. Besides, the prevalence of exploring Antibody therapeutics against AD, the shifts in distribution of “Biologic” candidates being investigated is largely parallel for both AD and PD.

### 6.4 Leveraging drug delivery vehicles for clinical translation

Figure 8a presents an alluvial flow diagram that illustrates the distribution of clinical trials for AD and PD intervening via the categorized therapy mechanisms, and additionally ties these trials to the delivery vehicle leveraged. Despite drug delivery technologies being heavily researched for the past several decades, it is evident that delivery vehicles are underutilized for clinical translation for both AD and PD. Clinical trials utilizing delivery vehicles only account for 1.07% and 4.99% of AD and PD clinical trials, respectively. There is a clinical need for additional research into the optimization and compatibility of delivery vehicles for neurodegeneration therapies. Delivery vehicles have the potential to increase the efficacy and efficiency of both existing and future AD and PD therapies.

Achieving target site‐specific accumulation of therapeutics is cumbersome, especially for neurological diseases given the multiple biological barriers such as the BBB and intraparenchymal diffusion.^125^ Advanced drug delivery vehicles of synthetic or biological nature may improve upon previously and currently explored therapeutics to provide greater accumulation in the brain to achieve therapeutic efficacy with reduced peripheral side effects.

The lack of a carrier severely limits the types of drugs that can cross the BBB, where more than 98% of small molecules and essentially 100% of biologics are incapable of bypassing an intact BBB.^126^ Drug delivery vehicles may improve brain targeting and expand the types of therapeutics that can achieve brain accumulation, which is naturally limited to small hydrophobic molecules.^73^, ^127^ For example, nanoparticle vehicle surface conjugation or direct modification of biologics with BBB‐targeting antibodies can leverage receptor mediated transcytosis across the BBB via transferrin, low‐density lipoprotein (LDL), and insulin receptors.^128^, ^129^ Drug incorporation into carriers also reduces systemic drug clearance and extends drug half‐life,^130^ increasing the opportunity for brain accumulation. In addition to further extending carrier half‐life,^131^ carrier surface functionalization with low affinity materials such as polyethylene glycol can also enhance diffusive ability, enabling the therapeutic to achieve a wider volume of distribution.^132^ Carrier shape can also affect neurovascular adhesion, as demonstrated by increased brain accumulation of rod‐shaped compared to spherical nanoparticles.^133^, ^134^ Furthermore, many free drugs that manage to cross the BBB are often readily ejected out of the brain parenchyma by several active efflux transporters including the P‐glycoprotein, ATP binding cassette, multidrug resistance‐associated proteins, and breast cancer resistance proteins (BCRP).^135^, ^136^ After entering the brain parenchyma, carriers may shield their drug cargo from efflux transporters expressed on the abluminal brain endothelium, reducing efflux transport out of the brain. Furthermore, cell therapies can deliver therapeutics or themselves act as the therapeutic^137^ and capitalize on intrinsic chemotactic capabilities to achieve targeted accumulation at injured brain regions following neuroinflammatory chemokine gradients.^138^

One of the challenges in translating drug delivery vehicle strategies to the clinic include an increased number of components for the therapeutic, which increases the regulatory hurdles for FDA approval. Delivery vehicles may also potentially involve more complexity leading to increased costs for manufacturing, involving multiple biomaterials and synthesis or conjugation steps. Developing cell biologics also entails additional considerations adding to production complexity, such as obtaining cells from autologous versus allogeneic sources and ex vivo cell culturing and modification. Nevertheless, considering there are no currently approved therapeutics that mitigate or stop neurodegeneration, the benefits of applying these novel delivery approaches may still outweigh the challenges towards translation. Furthermore, if these advanced drug delivery strategies prove successful, future manufacturing optimization and innovation could make these therapeutics more competitive for widespread treatment of neurodegenerative diseases. Fortunately, there is a growing percentage of trials utilizing delivery vehicles in phase 1 and 2 for both AD and PD among active clinical trials compared to past clinical trials (Figures 3c, 4c, 5c, and 6c), indicating increased adoption of drug delivery strategies.

## 7 NOVEL PRECLINICAL STRATEGIES FOR AD/PD TREATMENT

In addition to the expansion of using viral vectors and cell therapy including stem cells and various immune cells, there are other exciting strategies that are in their infancy of preclinical studies or barely entering clinical trials. One such strategy is the development of neurodegeneration vaccines, where the immune system is primed against Aβ or tau in AD,^139^ and against alpha‐synuclein in PD.^140^ Beyond conventional antigen/adjuvant strategies, Fessel imagines an mRNA vaccine strategy for AD, where mRNA self‐amplifies Aβ production.^141^ However, the efficacy and safety, in regards to autoimmunity, increased inflammation, and prematurely causing AD, has yet to be evaluated.^141^ With promise of CRISPR/Cas9 intervention in Huntington's disease, there is also exploration of CRISPR application in AD and PD.^142^, ^143^ CRISPR/Cas9 edited sRAGE‐MSCs reduced neuronal death and improved movement in a rotenone‐induced PD mouse model.^144^ Gyorgy et al. demonstrated deletion of APP Swedish mutation with CRISPR/Cas9 adeno‐associated viral vectors to reduce Aβ levels in transgenic mice overexpressing APP Swedish mutation.^145^ Another growing field of research is the role of extracellular vesicles (EVs), also known as exosomes or microvesicles depending on size, as biomarkers of disease progression in AD and PD.^146^, ^147^ EVs from various donor cell origins are also being explored as potential therapeutics due to their complex roles in cell‐to‐cell communication, and ability to cross the BBB and carry therapeutic cargo.^148^, ^149^ EVs derived from MSCs are of special interest as therapeutic carriers.^150^ EVs from human teeth stem cells administered intranasally indeed reduced gait impairments in a 6‐hydroxydopamine (6‐OHDA) rat model of PD.^151^ With the growing burden of AD and PD with no effective cures yet, novelty and innovation in therapeutic mechanism and delivery approaches is crucial to bringing effective treatments for neurodegenerative diseases to market.

## 8 CONCLUSION AND OUTLOOK

Clinicaltrials.gov has a wealth of information for gaining insight into the clinical frontier of any given disease. By meticulously organizing the data for AD and PD clinical trials, we have generated figures that succinctly provide information about these clinical trials across different clinical phases, therapy types, and time. This has enabled the illustration of meaningful trends in clinical trials over the years regarding the therapy mechanism of intervention, distribution of “Biologic” therapies explored, and growth of advanced drug delivery strategies. Although the scientific community has come a long way in terms of symptomatic management of AD and PD, therapeutic innovation in the field is branching into new directions to tackle root causes of disease progression. Notably, Anti‐aggregation therapies are taking over NTS therapies as the most common therapy mechanism. This is expected for AD but is also interestingly concurrently happening for PD. There is also a growing number of AD and PD clinical trials that are utilizing Anti‐inflammatory, Anti‐oxidant, and Regeneration strategies since 2011. The types of “Biologic” therapies used in the clinical frontier is also changing, with consistent use of Antibody‐, reduction of Natural product‐, and proteins/peptides‐based therapies, and increases in Cell therapy and Gene therapy approaches. There is also a growth in the use of delivery vehicles, although they are still heavily underutilized. Currently, many of the clinical trials investigating alternative therapeutic mechanism targets, types of “Biologic” therapies, and utilization of carriers are currently in phase 1 and 2 trials. The upcoming decades will bring many exciting advances as we witness which clinical trials survive running the clinical trial gauntlet to generate novel effective therapies on the market that can finally inhibit or possibly even cure AD and PD progression.

## AUTHOR CONTRIBUTIONS

**Puja Chopade:** Data curation (equal); formal analysis (equal); investigation (equal); methodology (equal); writing – original draft (equal). **Neha Chopade:** Data curation (equal); formal analysis (equal); investigation (equal); methodology (equal); writing – original draft (equal). **Zongmin Zhao:** Writing – review and editing (equal). **Samir Mitragotri:** Conceptualization (equal); resources (equal); supervision (equal); writing – review and editing (equal). **Rick Liao:** Conceptualization (lead); data curation (equal); formal analysis (equal); investigation (equal); methodology (equal); supervision (equal); validation (equal); visualization (lead); writing – original draft (lead). **Vineeth Chandran Suja:** Conceptualization (lead); data curation (equal); formal analysis (equal); investigation (equal); methodology (equal); supervision (equal); validation (equal); visualization (lead); writing – original draft (lead).

## CONFLICT OF INTEREST

The authors declare that they have no competing interests.

## Data Availability

All data generated or analyzed during this study are included in this published article.

## Appendix

**TABLE A1:** Definition of key terms

Term	Definition
Therapy mechanisms	
Neurotransmitter system	Therapies targeting neurochemical signaling between neurons.
Anti‐aggregation	Therapies targeting build of misfolded proteins such as the amyloid beta plaques.
Anti‐excitotoxicity	Therapies targeting excessive stimulation of neuronal receptors.
Anti‐inflammatory	Therapies targeting neuronal inflammation.
Anti‐oxidant	Therapies targeting imbalance between the neuronal production and clearance of reactive oxygen species.
Anti‐apoptotic	Therapies targeting programmed cell death.
Regeneration	Therapies targeting neurogenesis.
Administration route	
Intracerebral	Drug administration directly into the brain including intracisternal and intraventricular injections.
Intradermal	Drug administration via a superficial injection into the dermis.
Transdermal	Drug administration via absorption through the skin including using topical creams and patches.
Intestinal	Drug administration directly into the duodenum or upper jejunum.
Intraglandular	Drug administration directly into the glands including salivary glands.
Delivery vehicle	
Cellular	Drug delivery utilizing live cells including immune and stem cells
Viral	Drug delivery utilizing viral vectors.
Protein	Drug delivery utilizing protein‐based particles.
Outcome measures	
Psychiatric	Evaluation of cognitive and behavioral metrics using scales such as the Dementia Severity Rating Scale.
Biochemical	Concentration of biomarkers including enzymes such as glucocerebrosidase and proteins such as amyloid beta.
Pain	Evaluation of pain perception and nociceptive thresholds using tests such as thermotests.
Sleep	Evaluation of sleep quality and daytime sleepiness using metrics such as Epworth Sleepiness Scale and REM Sleep Behavior Disorders severity scale.
Volume	Evaluation of changes in hippocampus and total brain volume.
Motor	Evaluation of changes in movement using metrics such as Movement Disorders Society‐Unified Parkinson's Disease Rating Scale.
Pharmacokinetics	Evaluation of drug distribution within the body.
Gastrointestinal	Evaluation of gut health using metrics such as Bristol stool scale and Nepean dyspepsia index.
Safety	Evaluation of treatment‐emergent adverse events (TEAE's).
Fatigue	Evaluation of feeling of tiredness and weakness using metrics such as the Fatigue Severity Scale.
